# Differential Expression of *KCNJ12* Gene and Association Analysis of Its Missense Mutation with Growth Traits in Chinese Cattle

**DOI:** 10.3390/ani9050273

**Published:** 2019-05-24

**Authors:** Jie Cheng, Wenwen Peng, Xiukai Cao, Yongzhen Huang, Xianyong Lan, Chuzhao Lei, Hong Chen

**Affiliations:** College of Animal Science and Technology, Northwest A&F University, Yangling, Xianyang 712100, Shaanxi Province, China; chengjie1212@nwafu.edu.cn (J.C.); 18700481218@163.com (W.P.); cxkai0909@163.com (X.C.); hyzsci@126.com (Y.H.); lan342@126.com (X.L.); leichuzhao1118@126.com (C.L.)

**Keywords:** *KCNJ12*, SNP, myoblast differentiation, stature, Chinese cattle

## Abstract

**Simple Summary:**

A central goal of livestock genomic study is to find causal genes underlying economic traits and identify effective variations which can be used as molecular markers for livestock breeding. The cattle *KCNJ12* gene is an important candidate gene. To date, however, there have been no reports about the use of its missense mutation as a marker in cattle stature. In this study, missense mutation in *KCNJ12* was firstly verified, which led to a change in its protein sequence. Further, a significant association was detected between the mutation of *KCNJ12* and cattle stature, and we determined that the mutation in *KCNJ12* could be used as a molecular marker in beef breeding programs. In addition, expression analysis of the *KCNJ12* gene revealed high abundance in muscle and potential roles in bovine myocyte differentiation, which may be the subject of our future research.

**Abstract:**

The potassium inwardly rectifying channel, subfamily J, member 12 (*KCNJ12*) gene is a promising candidate for economic traits because of its crucial roles in myoblast development. Here, a missense mutation (Cys > Arg) was first detected to be located in exon 3 of *KCNJ12* from three Chinese cattle breeds by DNA-pool sequencing. Then, we performed an association analysis of this single-nucleotide polymorphism (SNP) with stature in three Chinese cattle populations (*n* = 820). A significantly positive correlation was revealed by a reduced animal general linear model and the CC genotype was the most favorable in three breeds. Further, we measured the expression profile of the *KCNJ12* gene in various cattle tissues and primary bovine skeletal muscle cells. Ubiquitous expression with high abundance in muscle was observed. Further, in primary bovine skeletal muscle cells, the *KCNJ12* mRNA expression was gradually up-regulated in differentiation medium (DM) compared with that in growth medium (GM), suggesting that the *KCNJ12* gene is involved in bovine myocyte differentiation. Conclusively, the *KCNJ12* gene is a functional candidate gene which can be used as a molecular marker for cattle breeding.

## 1. Introduction

Understanding the growth and development of skeletal muscle is one of the most important goals in animal and meat science. Meat characteristics are directly affected by many factors, among which genetic factors are of prime importance because genetic improvement is permanent and cumulative when inherited by subsequent generations. Genetic variation in livestock is known to be of the utmost importance. Therefore, identifying the causal loci of meat productivity and quality is a subject of intense research, and at present, only a fraction of these loci have been discovered [1]. Therefore, it is urgent to discover these loci and improve our understanding of their molecular mechanisms in skeletal muscle development.

Potassium inwardly rectifying channel, subfamily J, member 12 (*KCNJ12*) belongs to the inward-rectifier potassium channel family, which includes the strong inward-rectifier channels (Kir2.x), the G-protein-activated inward-rectifier channels (Kir3.x), and the ATP-sensitive channels (Kir6.x). The *KCNJ12* gene encodes an inwardly rectifying K^+^ channel protein, Kir2.2, and can combine with sulphonylurea receptors. The inwardly rectifying K^+^ channel can be blocked by divalent cations, and is one of the multiple inwardly rectifying channels contributing to the cardiac and nerve inward rectifier current (IK1) [2]. The inward-rectifier potassium channel, activated by phosphatidylinositol 4,5-bisphosphate, probably participates in controlling the resting membrane potential in electrically excitable cells and establishing the action potential waveform and excitability of neuronal and muscle tissues.

Motoneurons are important for regulating the function and properties of skeletal muscle. Potassium inward-rectifier (Kir) channels are important to establishing the resting membrane potential and regulating the muscle excitability. The *KCNJ12* gene is possibly involved in the regulation of muscle membrane properties and excitation–contraction coupling [3]. As might be expected, mutations in Kir channels can cause disorders affecting the heart and skeletal muscle, such as arrhythmia and periodic paralysis in humans [4]. For example, non-synonymous coding single-nucleotide polymorphisms (SNPs) of *KCNJ12* are associated with pathogenesis of rhabdomyosarcomas (RSCs) [5]. The expression level of *KCNJ12* is also relevant to dilated cardiomyopathy (DCM), and the number of Kir2.2 channels have been observed to be decreased in DCM ventricles [6]. Diseases associated with *KCNJ12* also include Smith–Magenis syndrome, among others [7].

Based on the genome-wide association study (GWAS) of copy number variations (CNVs) and growth traits in *Bos indicus*, Zhou (2016) has reported that *KCNJ12* could be a candidate gene for muscling through the modulation of muscle contraction and food intake [8]. Additionally, the two CNVs of *KCNJ12* were significantly associated with stature in four Chinese cattle populations, including NY (Nanyang cattle), JX (Jiaxian cattle), JA (Jian cattle), and GF (Guangfeng cattle) [9]. Although the *KCNJ12* gene located at *Bos taurus* autosome 19 (BTA19): 35,955,796–35,991,035 bp (AC_000176) has been widely proven as an important candidate for cattle stature [8,9], no reports on the SNP markers of the *KCNJ12* gene have been investigated in previous studies. Therefore, the aim of this study was to: (1) analyze the genetic polymorphisms of SNPs in *KCNJ12* using DNA-pool sequencing and forced PCR-RFLP (polymerase chain reaction-restriction fragment length polymorphism) in three Chinese cattle breeds, (2) establish the significant association between the mutation of the *KCNJ12* gene and cattle stature, and (3) examine the relative expression of the *KCNJ12* gene in different tissues as well as the time course of myoblast differentiation by RT-qPCR (reverse transcription-quantitative polymerase chain reaction). The results will provide new insights into the transcriptional regulation of the cattle *KCNJ12* gene and its potential applications in cattle breeding.

## 2. Materials and Methods

### 2.1. Ethics Statement

The protocols used in this study and for the animals were recognized by the Faculty Animal Policy and Welfare Committee of Northwest A & F University (FAPWC-NWAFU, protocol number, NWAFAC1008).

### 2.2. Animals and Data Collection

A total of 820 cows (*Bos taurus*) were used in this study, including Pinan cattle (PN, *n* = 372), Jin’nan cattle (JN, *n* = 205), and Xia’nan cattle (XN, *n* = 243), with PN, JN, and XN cattle each coming from three different farms, respectively. All samples collected for each breed were collected in the same season. The JN cattle is the main local cattle breed in China. PN and XN are well-known cultivated breeds, both of which have the features of superior growth and meat traits. Animals of each breed were selected to be unrelated for at least three generations to exclude sire effects. After weaning at 6 months of age, these animals were fed ad libitum under comfortable conditions (i.e., half grazing and half house feeding without straw cover). Records of cattle stature included: (1) withers height (WH), body oblique length (BOL), hip width (HW), chest girth (CG), thurl width (TW), and rump length (RL) in PN cattle; (2) withers height (WH), body oblique length (BOL), hip width (HW), paunch girth (PG), chest girth (CG), cannon bone circumference (CBC), and body weight (BW) in XN cattle; and (3) withers height (WH), body oblique length (BOL), hip width (HW), chest girth (CG), and rump length (RL) in JN cattle [10]. PN and JN groups were comprised of cows with different ages, and members of the XN group were adult cattle which were measured at 2 ± 0.2 years and were of different sexes. Detailed cattle record information is shown in Supplementary Table S1.

### 2.3. DNA Isolation and Genomic DNA Sequencing

Genomic DNA were extracted from 820 heparin-treated whole-blood samples and 30 muscle tissues collected from each individual of 30 XN cattle according to standard procedures [11]. The genomic DNA was diluted to 50 ng/µL, which was measured by spectrophotometer (1.6 < OD_260/230_ < 2.0 and 2.0 < OD_260/280_ < 3.0) and then stored at −80 °C [11]. Fifty DNA samples of each breed were randomly selected to construct three DNA pools, respectively. Based on the reference sequence in the NCBI database (GenBank accession no. AC_000176), eleven primers (P1–P11) were designed to screen variations in the exon of the *KCNJ12* gene (Supplementary Table S2) for PCR amplification from cattle genomic DNA, and PCR products were detected by 2% agarose gel electrophoresis. Then, the PCR products were sent to the sequencing company to complete the subsequent Sanger sequencing work in both directions (Shenggong, Shanghai, China).

### 2.4. Genotyping of Four Variations within Cattle KCNJ12 Gene

After screening the mutation, one pair of primers (Table 1) was redesigned to genotype the novel SNP (g.35989944T>C) for each individual. Given that the SNP locus has no natural restriction enzyme cutting sites, we mutated the primer to introduce a restriction enzyme cutting site. Therefore, we used a forced PCR-RFLP method to genotype the SNP. In detail, the *Pst I* site (CTGCA↓G) was created by changing the “AG” to “CT” in the reverse primer. After introducing the mismatch, the SNP could be genotyped by PCR products digested by *Pst I* [12]. The digested fragments were detected by electrophoresis of 3.5% agarose gel stained with nucleic acid dyestuff and using Marker Ⅰ, which included 100, 200, 300, 400, 500, and 600 bp bands.

### 2.5. Statistical Analysis

Population parameters such as genotypic frequencies, allele frequencies, homozygosity (Ho), heterozygosity (He), effective allele numbers (Ne), and polymorphism information content (PIC) were calculated using online software (http://www.msrcall.com/Gdicall.aspx). The χ^2^ tests for Hardy–Weinberg equilibrium (HWE) were calculated using PopGene software [13].

The association analysis was performed using a full animal general linear model (GLM), followed by a reduced statistical model, which was used in the final analysis. The full statistical model included fixed effects of genotype, sex, farm, and random effects of age. Association analysis between genotypes and cattle stature was performed in SPSS 18.0 (Statistical Product and Service Solutions, Version 18. 0 Edition, IBM, Armonk, NY, USA) using the following established reduced model after exclusion of non-significant confounders:

(1) $$Y_{ijlm}=u+A_{i}+G_{j}+S_{l}+e_{ijlm}$$

where $Y_{ijlm}$ is the observation of the cattle stature, $A_{i}$ is the random effect of age, $G_{j}$ is the fixed effect of genotype, $S_{l}$ is the fixed effect of sex, and $e_{ijlm}$ is the random residual error. For PN and JN cattle, S=0, and for XN cattle, A=0. Notably, the sire effect was not included in the model because these animals were unrelated for at least three generations. Multiple tests were corrected at *p* = 0.01 (*p* = 0.05/5) and *p* = 0.002 (*p* = 0.01/5) in JN, corrected at *p* = 0.0071 (*p* = 0.05/7) and *p* = 0.0014 (*p* = 0.01/7) in XN, and at *p* = 0.0083 (*p* = 0.05/6) and *p* = 0.0016 (*p* = 0.01/6) in PN.

### 2.6. Using PolyPhen to Predict if the Missense Mutation Altered the Protein of KCNJ12

PolyPhen (http://genetics.bwh.harvard.edu/pph2/) was used to determine how this missense mutation may alter the protein [14]. Mutations with their posterior probability scores associated with estimated false positive rates (FPRs) at or below the first (lower) FPR value are predicted to be “probably damaging” (more-confident prediction). Mutations with the posterior probabilities associated with false positive rates at or below the second (higher) FPR value are predicted to be “possibly damaging” (less-confident prediction). Mutations with estimated false positive rates above the second (higher) FPR value are classified as “benign”.

### 2.7. Cell Culture and Induction Differentiation

Primary bovine myoblasts were isolated from the bovine skeletal muscle of limbs of 90-day fetal cattle by enzyme digestion [15]. To promote myoblast proliferation, cells were reseeded in 12-well dishes and cultured in growth medium, which included high-glucose DMEM supplemented with 20% fetal bovine serum and double antibiotics (1% penicillin and streptomycin) at 37 °C under a 5% CO_2_ atmosphere.

To induce myoblast differentiation, when cells were at ~95% density marked as differentiation 0 day, the growth medium was replaced with 2% horse serum medium with 1% penicillin/streptomycin. The cells were refreshed with new medium every 24 h, and were cultured for −1, 0, 1, 2, and 4 days to induce differentiation prior to RNA extraction. The designation “−1 days” means the bovine myoblasts were in the proliferation phase with growth medium.

### 2.8. RNA Extraction, cDNA Synthesis, and Expression Analyses

To detect the expression analyses of *KCNJ12* in different tissues, total RNA was extracted from six tissues (i.e., heart, liver, spleen, lung, kidney, and muscle) collected from three 90-day-old fetuses (6 × 3 = 18 tissues).To detect the expression of *KCNJ12* with differentiating myoblasts, total RNA was extracted from differentiating myoblasts in differentiating myoblast day −1 (DMD−1), differentiating myoblast day 0 (DMD0), differentiating myoblast day 1 (DMD1), differentiating myoblast day 2 (DMD2), and differentiating myoblast day 4 (DMD4), respectively, and each period had three repeats. To reveal the correlations between genotype and expression level, 30 muscle samples were collected from each individual of 30 XN cattle, and were also used to extract RNA. Extraction of RNA using Trizol Reagent (TaKaRa, Kusatsu, Shiga Prefecture, Japan) followed the manufacturer’s protocol [16]. RNA integrity was assessed by electrophoresis on 1.0% agarose gel, and RNA purity was verified by measuring the absorbance at 260 and 280 nm using an ND-1000 spectrophotometer (NanoDrop Technologies, Wilmington, DE, USA). The cDNA for each sample was synthesized from an equal amount of total RNA (500 ng) using a PrimeScript RT reagent kit (TaKaRa, Kusatsu, Shiga Prefecture, Japan) following the manufacturer’s protocol.

The mRNA expression levels of *KCNJ12*, *MYOD*, *MYOG*, *MYHC*, and *GAPDH* were evaluated with a SYBR^®^ Premix Ex Taq™ kit (TaKaRa, Kusatsu, Shiga Prefecture, Japan) by qPCR in a Bio-Rad CFX96 RT-PCR System (Bio-Rad, Hercules, CA, USA). Templates were diluted to 50 ng/μL, and primers were diluted to 10 μM. Reaction mixtures totaling 13 μL contained 25 ng of genomic DNA or cDNA, 6. 5 μL SYBR^®^ Premix Ex Taq TM II (TaKaRa, Kusatsu, Shiga Prefecture, Japan), and 5 pmol of primers. The conditions for the thermal cycling test consisted of one 30 s cycle at 95 °C, followed by 39 cycles of 10 s at 95 °C, and 39 cycles of 30 s at 60 °C. Melting curves were completed at the end of the amplification with the conditions: 1 cycle at 95 °C for 1 min, then 1 cycle at 55 °C for 1 min, followed by a rate of increase of 0.5 °C/cycle from 55 to 95 °C. The primers were checked by melting curve analysis and no-template control reactions. The amplification efficiencies of all primers were measured with serial dilutions of cDNA (0.005, 0.05, 0.5, 5, 50, and 500 ng), and PCR efficiencies were similar for *KCNJ12*, *MYOD*, *MYOG*, *MYHC*, and *GAPDH* gene-specific primers (Table 2). The *GAPDH* gene was chosen as the internal reference gene for the qPCR analysis. The expression levels were calculated using 2^−∆∆Ct^. All experiments were repeated three times.

## 3. Results

### 3.1. Identification of Genetic Variation in the Cattle KCNJ12 Gene

**Figure 1:**
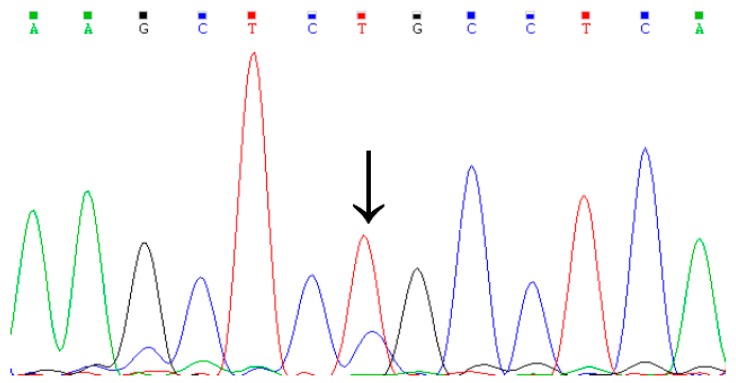
Sequencing result of the g.35989944T>C mutation in the *KCNJ12* gene.

**Figure 2:**
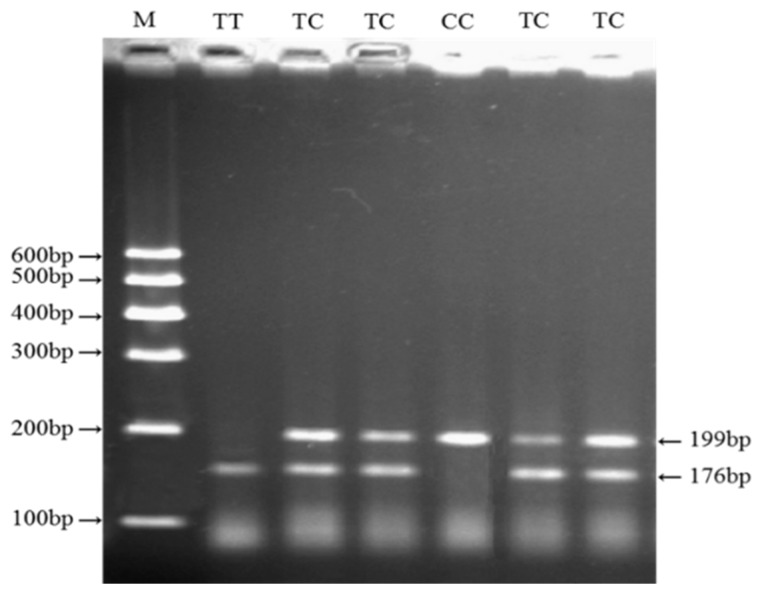
Polymerase chain reaction-restriction fragment length polymorphism (PCR-RFLP) of g.35989944T>C by agarose gel electrophoresis.

By DNA-pool PCR sequencing, one missense mutation in *KCNJ12*, named g.35989944T>C, was identified for the first time in three cattle breeds (Figure 1). The g.35989944T>C mutation was located in exon 3 (Cys > Arg) and had three genotypes based on the PCR products digested by *Pst I*. As shown in Figure 2, the genotypes were classified as CC (199 bp), TC (199 bp and 176 bp), and TT (176 bp) according to the agarose gel electrophoresis analysis.

### 3.2. Genotypes, Allele Frequencies, and Genetic Diversity of the SNP in the KCNJ12 Gene

The sample size, genotypic frequency, allelic frequency, homozygosity (Ho), heterozygosity (He), effective allele number (Ne), and polymorphism information content (PIC) of the SNP in the *KCNJ12* gene in the three cattle breeds are shown in Table 3. The results obtained from the preliminary analysis suggested that the frequencies of genotypes and alleles were different in the three cattle breeds. The results also indicated that the SNP was polymorphic and was in medium genetic diversity in all three cattle breeds (0.25 < PIC < 0.5). The χ^2^ test indicated that the SNP (g.35989944T>C) in JN cattle, a local breed, was in HWE (*p* > 0.05), but XN and PN cattle, two cultivated breeds, were not in HWE, given their different genetic backgrounds. A locus maintained in HWE suggests allelic balance during long evolution and breeding.

### 3.3. Association Study of g.35989944T>C with Cattle Stature

The results of the association analysis between the g.35989944T>C locus and cattle stature are shown in Table 4 (JN cattle), Table 5 (XN cattle), and Table 6 (PN cattle). For JN cattle, the SNP was found to be significantly associated with the WH and HW (*p* < 0.002). Notably, WH was higher for individuals with genotype CC (129.61 ± 0.55 cm) than TC (127.00 ± 0.73 cm) and TT (124.36 ± 1.69 cm). For XN cattle, the SNP was found to be significantly associated with CG (*p* < 0.0071), PG, and BW (*p* < 0.0014). BW was much higher for individuals with genotype CC (471.91 ± 10.42 cm) and TC (455.55 ± 7.24 cm) than TT (385.45 ± 12.52 cm). For PN cattle, the SNP was found to be markedly associated with the RL (*p* < 0.0083), BOL, HW, CG, and TW (*p* < 0.0016). BOL was higher for individuals with genotype CC (151.41 ± 1.24 cm) than TC (147.42 ± 0.72 cm) and TT (143.98 ± 1.32 cm).

### 3.4. Using PolyPhen to Determine if the Missense Mutation May Alter the Protein

In order to study if the missense mutation had effects on the protein of *KCNJ12*, we made a prediction using PolyPhen. The results are shown in Supplementary Figure S1. HumDiv is the preferred model for evaluating rare alleles, dense mapping of regions identified by genome-wide association studies, and analysis of natural selection. HumVar is the preferred model for diagnostics of Mendelian diseases which requires distinguishing mutations with drastic effects from all the remaining human variation, including abundant mildly deleterious alleles. This mutation was predicted to be “probably damaging”, with a score of 0.999 in HumDiv and it was predicted to be “probably damaging” with a score of 0.977 in HumVar. The KCNJ12 protein, called the inward-rectifier potassium channel Kir2.2, is also shown in Supplementary Figure S1 with phosphatidylinositol 4,5-bisphosphate (PIP2). The first pair, HumDiv, was compiled from all damaging alleles with known effects on the molecular function causing human Mendelian diseases, present in the UniProtKB database, together with differences between human proteins and their closely related mammalian homologs, assumed to be non-damaging. The second pair, HumVar, consisted of all human disease-causing mutations from UniProtKB, together with common human nsSNPs (MAF > 1%) without annotated involvement in disease, which were treated as non-damaging.

### 3.5. Expression Analyses of KCNJ12 in Cattle Tissues

In order to study if the variant named g.35989944T>C had an influence on mRNA expression levels, we first studied *KCNJ12* expression profiles in six tissues of 90-day old fetuses. After that, we performed the associations between *KCNJ12* genotypes and expression levels in muscle.

**Figure 3:**
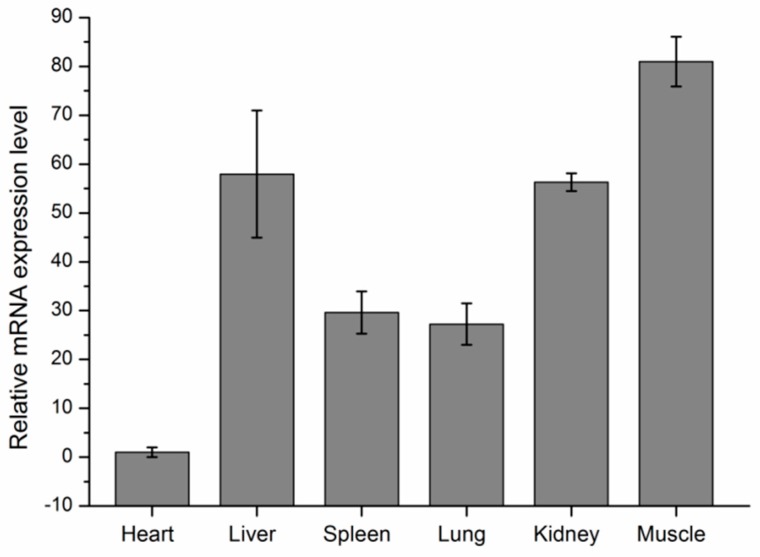
Expression profiling of *KCNJ12* in different tissues of three 90-day-old fetuses. The values are the averages of three samples calculated by 2^−∆∆Ct^. Error bars represent the standard error (SE) (*n* = 3) calculated by ∆Ct. The *GAPDH* gene was chosen as the internal reference gene for the qPCR analysis.

**Figure 4:**
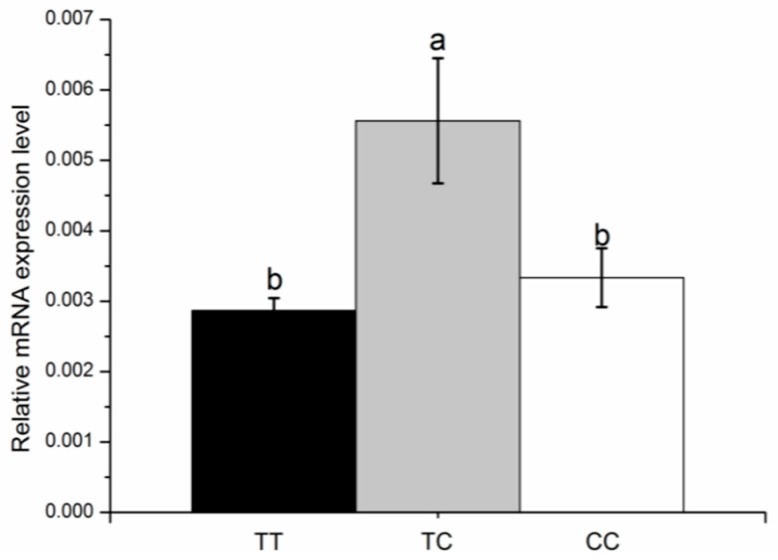
The effects of g.35989944T>C on *KCNJ12* gene expression in 30 muscle tissues. The *GAPDH* gene was chosen as the internal reference gene for the qPCR analysis. Thirty muscle samples were genotyped, including three TT samples, 15 TC samples, and 12 CC samples.

The expression levels of *KCNJ12* in different tissues were detected using RT-qPCR. As shown in Figure 3, the mRNA abundance of the cattle *KCNJ12* gene varied in different tissues, with the highest expression level in muscle. Because this study was concerned with the growth and development of muscle, 30 muscle samples of XN cattle were used to test the potential correlations between different genotypes and the *KCNJ12* gene mRNA expression level (Figure 4). We chose XN cattle given the following facts. Firstly, XN cattle was the first cultivated beef breed in China, which was a representative breed. Secondly, use of the XN cattle was promoted and they became widely distributed in China. Thirdly, sample size of XN cattle was larger than that of other breeds. Thirty muscle samples were genotyped, including three TT samples, 15 TC samples, and 12 CC samples. Given the limitations of the sample sizes, we only found that there was a tendency for the expression of heterozygous individuals with TC genotype to be higher than that in individuals with homozygous TT or CC genotypes.

### 3.6. KCNJ12 Expression during Primary Bovine Skeletal Muscle Cell Differentiation

**Figure 5:**
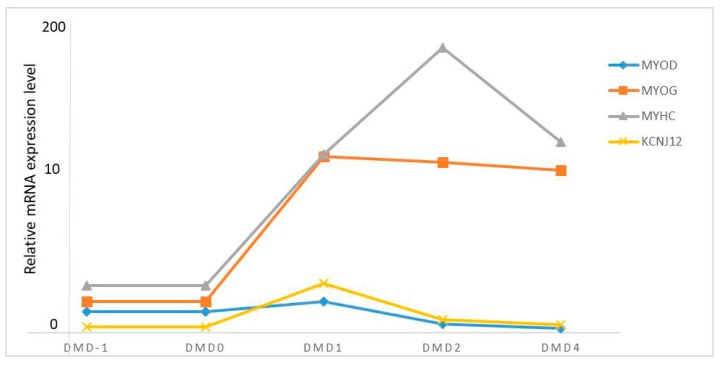
Expression of *KCNJ12*, *MYOD*, *MYOG*, and *MYHC* genes during myoblast differentiation. Relative mRNA expression levels of *KCNJ12*, *MYOD*, *MYOG*, and *MYHC* genes in different differentiation periods were analyzed by qPCR in primary bovine skeletal muscle cells. The mRNA expression levels of the four genes were normalized to *GAPDH*. The values 0–10 have the same unit as 10–200 on the Y-axis. DMD−1: differentiating myoblast day −1; DMD0: differentiating myoblast day 0; DMD1: differentiating myoblast day 1; DMD2: differentiating myoblast day 2; DMD4: differentiating myoblast day 4.

The expression levels of *KCNJ12*, *MYOD*, *MYOG*, and *MYHC* in primary bovine skeletal muscle cells serve as an excellent model system to study muscle cell differentiation in vitro [17]. Relative expression levels of these genes were detected during the differentiation of primary bovine skeletal muscle cells by RT-qPCR normalized to *GAPDH*. In order to represent the fold lines clearly in Figure 5, the unit of measurement on the *Y*-axis was altered, and the values 0–10 have the same distance as 10–200 on the *Y*-axis in Figure 5. DMD−1 refers to the bovine myoblasts in growth medium, and DMD1, DMD2, and DMD4 mean the bovine myoblasts were in differentiation medium. We found that the expression levels of the four genes were different on different differentiation days (Figure 5). The expression of *KCNJ12* was gradually up-regulated in differentiation medium (DM) compared with that in growth medium (GM), with a slight decrease after differentiation day 1 (DMD1), when myotubes were being formed. This was similar to the expression of *MYOD.* The relative expression of *MYOD* was greatest on DMD1. In addition, the relative expression of *MYOG* was very high for DMD1, DMD2, and DMD4, and the peak value of *MYHC* appeared on the second day of differentiation (DMD2).

## 4. Discussion

We are in a phase of unprecedented progress in identifying genetic loci that cause variations in the economic traits of livestock, and more markers are required for the implementation of genomic selection in Chinese cattle [18]. In this study, we identified one SNP—a missense mutation of the bovine *KCNJ12* gene—through DNA pool sequencing in three Chinese cattle breeds. The SNP in the *KCNJ12* gene has abundant genetic diversity, which may be essential for production improvement. Recently, a considerable body of literature has grown up around the theme that missense mutations can influence gene expression [19] and protein function [20,21]. The SNP in *KCNJ12* is a missense mutation located in exon 3 of the bovine *KCNJ12* gene, and may affect translation efficiency, thereby altering the function of the *KCNJ12* protein. Association analysis indicated that the SNP was significantly associated with cattle stature by using 820 cattle samples (PN, *n* = 372; JN, *n* = 205; XN, *n* = 243). In fact, larger sample sizes may allow for more accurate results, but because cattle are large domestic animals unlike sheep, goats, chickens, pigs, etc., it is not easy to obtain large sample sizes. Previous work has shown that the sample sizes available in this study are sufficient to draw preliminary conclusions [22,23,24]. We demonstrated that the CC genotype was superior for the growth traits measured in this study. This result suggests a breeding strategy whereby cattle with the CC genotype are selected to produce offspring. There are no annotated genes (UCSC) for around 77 kb upstream of the bovine *KCNJ12*, and for about 92 kb downstream. According to the existing research reports, the linkage imbalance intensity decreases with the increase of genomic distance, and R^2^ < 0.1 when the distance between two points is more than 50 kb. Therefore, we did not conduct a linkage imbalance analysis in a different gene. Moreover, accurate linkage imbalance analysis requires population-based genome resequencing, which is very complicated and necessitates the collection of many samples.

The mRNA level which occurs after transcription and before translation indicates the relationship between DNA and protein, so mRNA levels are usually measured to investigate the role of target genes [17]. Expression analysis of the bovine *KCNJ12* gene showed that *KCNJ12* was widely expressed in different tissues, and was particularly highly expressed in muscle (Figure 3), which implies that the *KCNJ12* gene may play a major role in cattle muscle development. Besides, it can be seen from the data in Figure 4 that the relative expression of the TC genotype was the highest among three genotypes, but the CC genotype had better growth performance. Results indicate that there is complex post-transcription regulation of *KCNJ12*. As determined by PolyPhen software, SNPs may affect the structure of the protein, and may thus affect the phenotype (Supplementary Figure S1). It may be that the amino acid change had a significant effect on protein formation, or that the mutation had an effect such as linkage with another key mutation. For example, Q320P is a major QTN with a relatively stable influence on litter size, and V397I may have a significant effect on litter size due to linkage with the Q320P mutation [25]. These results are preliminary, and therefore further investigations are urgently needed to uncover the genetic causality of *KCNJ12* behind the significant association.

Muscle progenitor cells differentiate into myoblasts through proliferation, differentiation, and fusion into multinucleated myotubes, eventually forming mature muscle fibers [26,27]. Muscle development is mainly regulated by a series of transcription factors which include paired box protein 3/7 (Pax3/7), the myogenic regulator (MRF) family (MYOD, myogenin, Myf5, and MRF4) [28,29], and the myocyte enhancer factor 2 (MEF2) family (MEF2A, MEF2B, MEF2C, and MEF2D) [26,30,31]. In addition, muscle development is also directly or indirectly regulated by other protein-coding genes. Many published studies have reported that the *KCNJ12* (potassium inwardly rectifying channel) gene, as the name suggests, is crucial in the transmission of nerve impulses. Motoneurons are important for regulating the function and properties of skeletal muscle, so the gene may be involved in the regulation of muscle development [32]. In our study, as can be seen from Figure 5, the highest expression of *KCNJ12* was at DMD1, which was similar to *MYOD*. The gene was up-regulated in differentiation medium (DM) compared with levels seen in growth medium (GM). Myotubes were being formed, which indicates that *KCNJ12* plays an important role in the differentiation of bovine skeletal muscle. Moreover, the highest expression of *MYOD* was at differentiation day 1 (DMD1). The relative expression of *MYOG* was very high for DMD1, DMD2, and DMD4, and the peak value of *MYHC* appeared on the second day of differentiation (DMD2). Therefore, we can deduce that cattle genes *KCNJ12*, *MYOD*, *MYOG*, and *MYHC* were activated in different stages. This result is also consistent with a previous study indicating that *MYOD* can influence muscle differentiation in its pre-differentiation period, and even in post-proliferation, and *MYOG* and *MYHC* were found to influence post-differentiation in C2C12 cells [33]. These data strongly suggest that *KCNJ12* polymorphisms could be used as a molecular marker for marker-assisted selection in cattle breeding.

## 5. Conclusions

An association analysis was conducted between the mutation of *KCNJ12* and cattle stature in a beef breeding program, and the CC genotype of g.35989944T>C was the most favorable genotype in all three breeds. The expression analysis of *KCNJ12* gene revealed high abundance in muscle and potential roles in bovine myocyte differentiation. This study will provide some useful information for cattle breeding.

## Figures and Tables

**Table 1:** Polymerase chain reaction (PCR) primer sequences and approach for the identification of single-nucleotide polymorphism (SNP) in *KCNJ12*.

Name	Chr. Position	Primer Sequences (5′–3′)	Genotyping Method	Tm (°C)	Restriction Enzyme	Genotype Pattern (bp)
SNP	g.35989944 T>C	F: CGAGGAGTGCCCGGTGGCGGTGTTCAT	PCR-RFLP	57	*Pst I*, CTGCA↓G	199 (176 + 23)
		R: TAGGTTGCCCACGCGCCACATG***CT***GC				

The italic and bold in primer sequences indicates the introduction of a mismatch.

**Table 2:** PCR primer sequences of *KCNJ12* and *GAPDH* gene in cattle for quantitative polymerase chain reaction (qPCR).

Gene Name	Primer Sequences (5′–3′)	Primer Efficiencies	Annealing Temp. (°C)
*MyoD*	F: ACGGCATGATGGACTACAGC	97.2%	60
	R: AGGCAGTCGAGGCTCGACA		
*MyoG*	F: CAAATCCACTCCCTGAAA	98.7%	60
	R: GCATAGGAAGAGATGAACA		
*MyHC*	F: TGCTCATCTCACCAAGTTCC	98.3%	60
	R: CACTCTTCACTCTCATGGACC		
*KCNJ12*	F: TGGGCAACCTACGCAAGAGC	97.9%	60
	R: GCAGGATGGTGATGGGAGACA		
*GAPDH*	F: CGACTTCAACAGCGACACTCAC	97.6%	60
	R: CCCTGTTGCTGTAGCCAAATTC		

**Table 3:** Population genetic indices of the SNP mutation.

Name	Breeds (Sizes)	Genotype Freq.			Allele Freq.		HWE-χ^2^	*p*	Ho	He	Ne	PIC
		TT	TC	CC	T	C						
SNP	XN/243	0.082	0.506	0.412	0.335	0.665	4.455	<0.05	0.554	0.446	1.804	0.346
g.35989944	JN/205	0.063	0.293	0.644	0.210	0.790	2.813	>0.05	0.668	0.332	1.496	0.277
T>C	PN/372	0.167	0.602	0.231	0.468	0.532	16.301	<0.05	0.502	0.498	1.992	0.374

PIC: polymorphism information content; PIC < 0.25: low polymorphism; 0.25 < PIC < 0.5: intermediate polymorphism; PIC > 0.5: high polymorphism. Freq.: Frequencies; He: heterozygosity; Ho: homozygosity; HWE: Hardy–Weinberg equilibrium; Ne: effective allele number; XN: Xia’nan cattle; JN: Jin’nan cattle; PN: Pinan cattle.

**Table 4:** Association between the *KCNJ12* variation and cattle stature in Jin’nan (JN) cattle.

Locus	Genotypes	Body Trait (cm, Mean ± SE)				
		Withers Height	Hip Width	Body Oblique Length	Chest Girth	Rump Length
g.35989944 T>C	CC	129.61 ^A^ ± 0.55	132.02 ^A^ ± 0.65	153.26 ± 0.95	186.74 ± 1.13	48.63 ± 0.34
	TC	127.00 ^B^ ± 0.73	129.50 ^B^ ± 0.92	151.41 ± 1.51	181.82 ± 2.25	47.34 ± 0.80
	TT	124.36 ^B^ ± 1.69	125.36 ^B^ ± 2.02	146.09 ± 3.16	176.64 ± 4.36	46.18 ± 1.43
	*p*	0.001	0.002	0.074	0.013	0.061

Different letters in the same column indicate significant difference (A,B: *p* < 0.002). SE: standard error.

**Table 5:** Association between the *KCNJ12* variation and cattle stature in Xia’nan (XN) cattle.

**Locus**	**Genotypes**	**Body Trait (Mean ± SE)**			
		**Withers Height (cm)**	**Hip Width (cm)**	**Body Oblique Length (cm)**	**Chest Girth (cm)**
g.35989944T>C	CC	129.07 ± 0.73	137.86 ± 0.63	151.31 ± 1.53	189.32 ^a^ ± 1.45
	TC	129.26 ± 0.92	135.58 ± 0.83	148.52 ± 4.61	185.16 ^a^ ± 2.42
	TT	127.00 ± 1.02	135.63 ± 1.60	148.45 ± 0.22	176.55 ^b^ ± 2.54
	*p*	0.383	0.246	0.751	0.003
**Locus**	**Genotypes**	**Body Trait (Mean ± SE)**			
		**Paunch Girth (cm)**	**Cannon Bone CRCM (cm)**		**Body Weight (kg)**
g.35989944T>C	CC	220.98 ^A^ ± 2.06	18.11 ± 0.28		471.90 ^A^ ± 10.42
	TC	217.60 ^A^ ± 1.54	18.51 ± 0.18		455.55 ^A^ ± 7.24
	TT	205.64 ^B^ ± 3.22	18.00 ± 0.39		385.45 ^B^ ± 12.52
	*p*	0.001	0.467		<0.001

Different letters in the same column indicate significant difference (a,b: *p* < 0.0071; A,B: *p* < 0.0014). CRCM: circumstances; SE: standard error.

**Table 6:** Association between the *KCNJ12* variation and cattle stature in Pinan (PN) cattle.

**Locus**	**Genotypes**	**Body Trait (cm, Mean ± SE)**		
		**Withers Height**	**Body Oblique Length**	**Hip Width**
g.35989944T>C	CC	126.70 ± 0.75	151.41 ^A^ ± 1.24	133.37 ^A^ ± 0.74
	TC	124.59 ± 0.41	147.42 ^B^ ± 0.72	131.41 ^B^ ± 0.40
	TT	122.59 ± 0.70	143.98 ^C^ ± 1.32	129.46 ^C^ ± 0.69
	*p*	0.04	<0.001	0.001
**Locus**	**Genotypes**	**Body Trait (cm, Mean ± SE)**		
		**Chest Girth**	**Thurl Width**	**Rump Length**
g.35989944T>C	CC	178.39 ^A^ ± 1.38	47.05 ^A^ ± 0.38	49.35 ^a^ ± 0.40
	TC	172.91 ^B^ ± 0.88	45.43 ^B^ ± 0.28	48.28 ^b^ ± 0.26
	TT	169.22 ^B^ ± 1.72	44.57 ^B^ ± 0.52	47.31 ^b^ ± 0.49
	*p*	<0.001	0.001	0.008

Different letters in the same column indicate significant difference (a,b: *p* < 0.0083; A,B,C: *p* < 0.0016). SE: standard error.

## Supplementary Materials

The following are available online at https://www.mdpi.com/2076-2615/9/5/273/s1, Table S1: Detailed information about cattle records, Table S2: Primer information for the PCR amplification of the bovine KCNJ12 gene, Figure S1: This missense mutation probably alters the protein (as determined using PolyPhen).

## Abbreviations

BOL: body oblique length; BW: body weight; CBC: cannon bone circumference; CG: chest girth; CNV: copy number variation; DCM: dilated cardiomyopathy; DMD: differentiating myoblast day; FAPWC-NWAFU: Faculty Animal Policy and Welfare Committee of Northwest A&F University; GF: Guangfeng cattle; GWAS: genome-wide association study; HW: hip width; HWE: Hardy–Weinberg equilibrium; IK1: cardiac and nerve inward rectifier current; JA: Jian cattle; JN: Jin’nan cattle; JX: Jiaxian cattle; KCNJ12: potassium inwardly rectifying channel, subfamily J, member 12; Kir: potassium inward-rectifier; Kir2.x: strong inward-rectifier channel; Kir3.x: G-protein-activated inward-rectifier channel; Kir6.x: ATP-sensitive channel; NY: Nanyang cattle; PCR-RFLP: polymerase chain reaction-restriction fragment length polymorphism; PG: paunch girth; PIP2: phosphatidylinositol 4,5-bisphosphate; PN: Pinan cattle; qPCR: quantitative polymerase chain reaction; QTL: quantitative trait loci; RSC: rhabdomyosarcoma; RL: rump length; RT-PCR: reverse transcription PCR; SNP: single-nucleotide polymorphism; TW: thurl width; WH: withers height; XN: Xia’nan cattle.
