# Supplementary material for: Differential Expression of KCNJ12 Gene and Association Analysis of Its Missense Mutation with Growth Traits in Chinese Cattle
Source: Animals (Basel). 2019 May 24;9(5):273. doi: 10.3390/ani9050273 (PMC6562504; doi:10.3390/ani9050273)
Supplement: Supplementary file 1 [file animals-09-00273-s001.pdf]

Supplementary files: Differential Expression of KCNJ12 Gene and Association Analysis of Its Missense Mutation with Growth Traits in Chinese Cattle

Table S1. Detailed information about cattle records.

Breeds	Housed	Collected Record					
		Ages (years)			Sex		
		≤1.5	2–3	≥3.5	Sire	Bullock	Dam
PN (*n* = 372)	Yes	0	278	94	0	0	372
JN (*n* = 205)	Yes	0	180	25	0	0	205
XN (*n* = 243)	Yes	0	243	0	16	30	197

PN: Pinan cattle; JN: Jin'nan cattle; XN: Xia'nan cattle.

Table S2. Primer information for the PCR amplification of the bovine *KCNJ12* gene.

Primer Name	Primer Sequences (5′–3′)	Position (AC_000176)	Amplicon Size (bp)
P1	F: CATAGGCTGCTGGTGGGT<br>R: GGAGGGAAGAGGCTCAGTT	35,953,344–<br>35,953,813	470
P2	F: CCATTTGCTGGAACTGTCTC<br>R: CCAGTCTGAACCCTTGCTC	35,953,663–<br>35,954,559	897
P3	F: AAGCCATCCTCCTTGGTTAGA<br>R: GGACTCCTTTCCCGTTGC	35,954,370–<br>35,954,917	548
P4	F: GAGGAGCACAGAAAGAAAGCA<br>R: GGGGAGAAAGGGGAGAAAA	35,954,788–<br>35,956,017	1230
P5	F: AGCCTGGGATCAGATAGCAGC<br>R: CACTCGCCAGCGGAGAACA	35,955,547–<br>35,956,420	874
P6	F: GCTCCGACTTCATAGGGT<br>R: TGGGTGGTCAAGCGTGTA	35,982,793–<br>35,983,704	912
P7	F: GATGGAGGCGTGGAGGTT<br>R: CGTCTTGTGGAAATGCGAGT	35,989,184–<br>35,990,360	1177
P8	F: GTCACGGAGGAGGGCGAGTA<br>R: CGGCACCATCAGGCACAT	35,990,025–<br>35,991,193	1169
P9	F: TTCGGCTTTCTCAATCTTAG<br>R: GTTCAGCATCAGGGCATAG	35,990,978–<br>35,991,792	815
P10	F: CACAGGCTTGTTCTTGATGG<br>R: GGGCACTAAAGAGGGAGAC	35,991,909–<br>35,992,711	803
P11	F: AAAGGGTCAATCCCAAGC<br>R: TCCAGGAAAAGCCGTCAC	35,992,251–<br>35,993,157	907

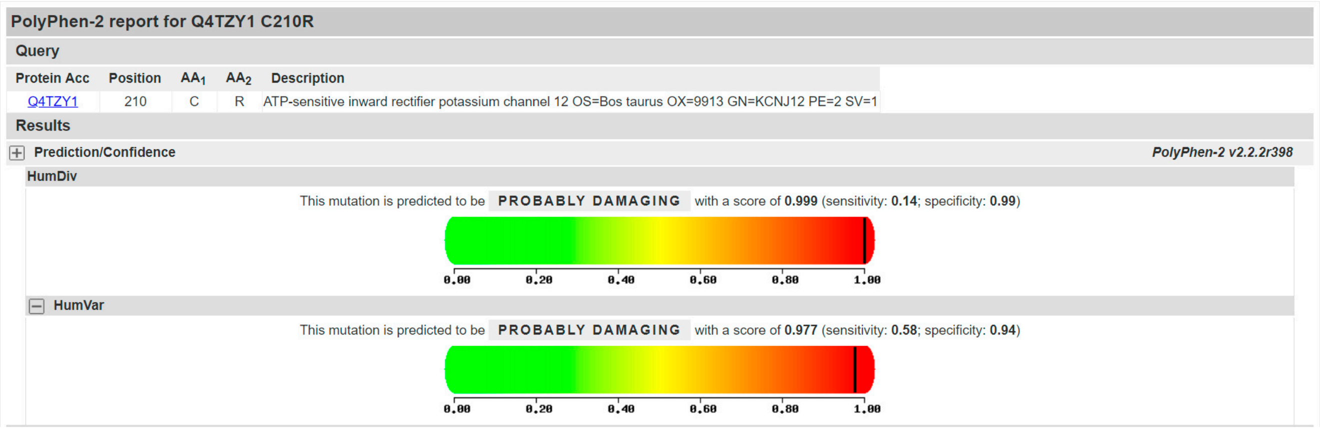

(A)

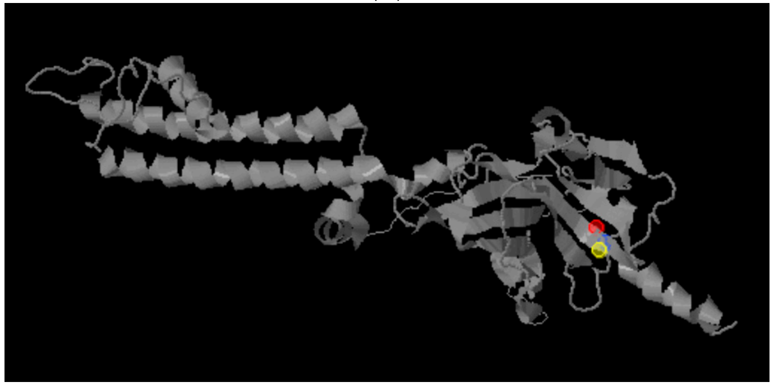

(B)

Figure S1. This missense mutation probably alters the protein (as determined using PolyPhen). (**A**) This mutation is predicted to be PROBABLY DAMAGING with a score of 0.999 in HumDiv; and this mutation is predicted to be PROBABLY DAMAGING with a score of 0.977 in HumVar. (**B**) Inward-rectifier potassium channel Kir2.2 in complex with PIP2. The colorful circles are residue 210 (Cys>Arg), which was an SNP at the DNA level.

© 2019 by the authors. Licensee MDPI, Basel, Switzerland. This article is an open access article distributed under the terms and conditions of the Creative Commons Attribution (CC BY) license (http://creativecommons.org/licenses/by/4.0/).
